# Investigating and Modulating Physiological and Pathological Brain Oscillations: The Role of Oscillatory Activity in Neural Plasticity

**DOI:** 10.1155/2019/9403195

**Published:** 2019-11-26

**Authors:** Andrea Guerra, Matteo Feurra, Giovanni Pellegrino, John-Stuart Brittain

**Affiliations:** ^1^IRCCS Neuromed, Pozzilli, Italy; ^2^National Research University, Higher School of Economics, Moscow, Russia; ^3^Centre for Cognition and Decision-Making, Institute for Cognitive Neuroscience, National Research University, Higher School of Economics, Moscow, Russia; ^4^Department of Neurology and Neurosurgery, Montreal Neurological Institute and Hospital, McGill University, Montréal, Canada; ^5^School of Psychology, University of Birmingham, Birmingham, UK

There is accumulating evidence that oscillatory activity plays a significant role in regulating brain function. Rhythmic phenomena are routinely observed during perception, motor, and cognitive tasks and have been implicated in altered functions across a broad range of diseases [1]. Several studies suggest that the alpha rhythm gates information flow, beta inhibits changes in motor activity and is responsible for the maintenance of the current sensorimotor or cognitive state, and gamma reflects intracortical local synchronization [1–3]. However, so far, understanding of the contribution of these rhythms to human behaviour and the manifestation of symptoms in disease states is limited. Moreover, the relationship between brain oscillations and neural plasticity is not clear, although recent evidence supports a link. For instance, it has been demonstrated that resonant rhythms in sensorimotor areas modulate motor learning and enhanced high-gamma activity in the primary motor cortex influences LTP/LTD-like plastic mechanisms [1, 2, 4]. As such we find ourselves in an era where we are rapidly garnering the tools to not only observe brain activity but also alter neural processes in a circumscribed manner. Such causal interactions allow deeper understanding of the role of neural oscillations in everyday life and of how changes in rhythmic activity can lead to altered functions in disease states. Noninvasive electrophysiological techniques such as high-density EEG and magnetoencephalography (MEG), invasive recordings of local field potentials, and advanced neuroimaging techniques able to infer brain oscillatory activity are now increasingly combined with different forms of brain stimulation [5, 6]. Sensory rhythmic stimulation, transcranial magnetic stimulation (TMS), and transcranial alternating current stimulation (tACS), used alone or in combination, allow targeting and causally interacting with rhythmic brain activity [2–4, 7]. Also, EEG activity can be used to guide TMS in a closed-loop configuration so as to induce and/or interfere with specific brain states [8]. These novel approaches provide new opportunities for drawing strong parallels between oscillatory activity and brain functions, including processes of cortical plasticity.

In the current special issue, we present ten articles that contribute to the debate on brain oscillations, plasticity, and behaviour. These articles address many aspects of brain oscillations, ranging from innovative methodologies to the rhythmic modulation of physiological function, the characterization of cortical and subcortical electrophysiological changes in the pathophysiology of neurological and psychiatric disorders, as well as the study of neurophysiological substrates of therapies that reflect neural plastic changes. Finally, specific patterns of brain oscillations have been proposed as possible biomarkers to drive treatments or predict clinical outcomes in neurological disorders.

At a systems level, S. Glim et al. showed that by adopting a TMS-EEG coregistration technique, single-pulse and repetitive TMS can transiently enhance theta-gamma, alpha-gamma, and beta-gamma phase-amplitude coupling (PAC). The demonstration of the effectiveness of this perturbational approach paves the way for interventions to study and modulate PAC in different physiological and pathological conditions. A. Lehr et al. applied tACS at theta frequency to the left dorsolateral prefrontal cortex in young healthy subjects during a color-word Stroop task in order to investigate cognitive control and conflict detection processes that involve prefrontal brain regions. The study revealed a reduction of the Stroop effect (response time difference between congruent and incongruent trials) and a modulation of adaptive mechanisms of the cognitive control network, providing evidence for a causal role of theta oscillations in cognitive control. The role of brain rhythms on physiological function was also explored by S. Ricci et al. through the use of high-density EEG recordings. In line with previous reports [9], they found that elderly participants and young subjects share similar patterns of cortical oscillations. In particular, the authors demonstrated that the depth of beta modulation in the sensorimotor and frontal regions increases during practice, an expression of cortical plastic mechanisms, and this change occurs regardless of age.

Specific alterations in oscillatory activity occur in pathological conditions. A. Ahnaou et al. demonstrated robust functional changes in the hippocampus of a tau-seeding mouse model in which preformed synthetic tau fibrils were injected into the locus coeruleus. Alterations in the functional properties of the network included impaired coherence and global coherent network efficiency in the ipsilateral hippocampus, deficient propagation directionality of gamma oscillations, and decreased theta-gamma PAC in the hippocampal circuit, suggesting early dysfunctional hippocampal networks and impaired synaptic plasticity in brainstem tau pathology. C. Jiang et al. show that neuropathological mechanisms contributing to cancer-related fatigue include a reduced corticomuscular coherence in alpha and beta frequency bands. Q. Ye et al. examined EEG recordings of spontaneous fluctuations of neuronal oscillations and functional connectivity during resting state in patients with somatoform pain disorder. The enhancement of parietal alpha rhythm, positively correlating with the somatization severity, as well as the increase in alpha frontoparietal connectivity, were suggested as pathophysiological hallmarks of processes underlying somatoform pain.

The study of brain oscillations also guides medical treatments and can even help to identify neural substrates for therapeutic approaches. Indeed, Z. Shen et al. observed that the effects of electroacupuncture in regulating pain memory-related behaviours in rats are paralleled by a downregulation of theta power in the rostral anterior cingulate cortex; these neurophysiological changes are therefore associated with the inhibited retrieval of aversive memories and the alleviation of pain memory-induced aversive behaviours. R. I. Carino-Escobar et al. conducted a longitudinal analysis of changes in brain rhythms by means of EEG recordings in subacute stroke patients during an innovative interventional strategy for hand rehabilitation. Event-related (de-)synchronization (ERD/ERS) in alpha and beta were modified across the intervention sessions, suggesting neural plastic phenomena, with the latter predicting the extent of upper limb motor recovery.

The study of brain oscillations also allows identifying specific and reliable patterns of activity that can be used as biomarkers and then applied in clinical practice. In this regard, the clinical trial from D. Güntensperger et al. conducted in patients suffering from chronic tinnitus took advantage of an adjusted alpha/delta neurofeedback protocol based on the measurement of individual alpha peak frequency, instead of the use of a fixed alpha frequency. This protocol led to a sustained reduction of tinnitus-related distress and to a temporary effect on tinnitus loudness. Finally, F. Zappasodi et al. followed up 120 patients affected by a monohemispheric stroke in the territory of the middle cerebral artery. They demonstrated that, in the acute phase, the bilateral, ipsi-, and contralesional increase in low band activity (i.e., increase in interhemispheric symmetry of the homologous areas' powers) predicted a reduced functional outcome in the subsequent stabilized phase. Interestingly, this biomarker, based on pathological changes in cortical oscillatory activity, could be used for patient selection in designing rehabilitation treatments and/or noninvasive neuromodulation protocols [10].

The achievements of the presented research allow us to confirm that brain oscillations play an important role in driving physiological brain function, as well as dysfunction in pathological conditions. Neural plastic processes appear to be both influenced, and indexed by, changes in power and connectivity. The impressive array of neurophysiological tools now at our disposal (including high-density EEG, MEG, TMS-EEG, and tACS) allow us to study and modulate oscillatory activities in increasingly more prescriptive ways. This level of observation coupled with intervention allow us to draw strong parallels between brain oscillatory function and behaviour, including symptomology. We hope that by continuing to clarify the role of brain rhythms in physiological and pathological conditions, we can facilitate the identification of neurophysiological biomarkers for clinical utility, and that subsequent manipulation of brain state through optimised forms of neural intervention will induce long-term plastic changes capable of maintaining prolonged amelioration of symptoms. We see strong evidence that this approach is not only feasible but also imminently achievable, and we encourage ongoing research in this field.

## References

[1] Giovanni A., Capone F., di Biase L. (2017). Oscillatory activities in neurological disorders of elderly: biomarkers to target for neuromodulation. *Frontiers in Aging Neuroscience*.

[2] Guerra A., Suppa A., Bologna M. (2018). Boosting the LTP-like plasticity effect of intermittent theta-burst stimulation using gamma transcranial alternating current stimulation. *Brain Stimulation*.

[3] Pellegrino G., Arcara G., Di Pino G. (2019). Transcranial direct current stimulation over the sensory-motor regions inhibits gamma synchrony. *Human Brain Mapping*.

[4] Guerra A., Suppa A., Asci F. (2019). LTD-like plasticity of the human primary motor cortex can be reversed by *γ*-tACS. *Brain Stimulation*.

[5] Thut G., Bergmann T. O., Fröhlich F. (2017). Guiding transcranial brain stimulation by EEG/MEG to interact with ongoing brain activity and associated functions: a position paper. *Clinical Neurophysiology*.

[6] Pellegrino G., Maran M., Turco C. (2018). Bilateral transcranial direct current stimulation reshapes resting-state brain networks: a magnetoencephalography assessment. *Neural Plasticity*.

[7] Shpektor A., Nazarova M., Feurra M. (2017). Effects of transcranial alternating current stimulation on the primary motor cortex by online combined approach with transcranial magnetic stimulation. *Journal of Visualized Experiments*.

[8] Zrenner C., Belardinelli P., Müller-Dahlhaus F., Ziemann U. (2016). Closed-loop neuroscience and non-invasive brain stimulation: a tale of two loops. *Frontiers in Cellular Neuroscience*.

[9] Ferreri F., Vecchio F., Guerra A. (2017). Age related differences in functional synchronization of EEG activity as evaluated by means of TMS-EEG coregistrations. *Neuroscience Letters*.

[10] Guerra A., López-Alonso V., Cheeran B., Suppa A. (2017). Solutions for managing variability in non-invasive brain stimulation studies. *Neuroscience Letters*.

